# Internal hernia from the interureteric space after robot-assisted radical cystectomy and urinary diversion: Two case reports

**DOI:** 10.1097/MD.0000000000017222

**Published:** 2019-10-11

**Authors:** Li-Hsien Tsai, Wei-Juan Li, Guang-Heng Chen, Po-Fan Hsieh, Chao-Hsiang Chang

**Affiliations:** Department of Urology, China Medical University Hospital, North District, Taichung, Taiwan.

**Keywords:** internal hernia, radical cystectomy, urinary diversion

## Abstract

**Rationale:**

Radical cystectomy and urinary diversion remains the definite management for muscle invasive bladder urothelial cancer. Internal herniation caused by ureteral adhesion is an extremely rare complication after the procedure. To the best of our knowledge, this is the first case report of small bowel obstruction and internal herniation occurring between bilateral ureters and urinary diversion after robot-assisted radical cystectomy (RARC).

**Patient concerns:**

A 64-year-old woman presented with symptom of small bowel obstruction such as nausea, vomiting, and abdominal fullness after RARC and Indiana pouch. Another 61-year-old man presented with left obstructive hydronephrosis and recurrent pyelonephritis after RARC and ileal conduit.

**Diagnosis:**

Both patients received computed tomography scans and the results were suggestive of small bowel herniation between bilateral ureters and urinary diversion.

**Interventions:**

The 2 patients underwent open ureterolysis and internal hernia reduction. During the operation, bowel loop herniation between the interureteral spaces were found.

**Outcomes:**

Both patients recovered smoothly after second operation.

**Lessons:**

The incidence of internal herniation may increase by the growing use of RARC. Suitable stoma position, appropriate length of ureter dissection, and retroperitonealization can help prevent this complication.

## 1 Introduction

Radical cystectomy with urinary diversion has been considered as one of the most challenging procedures in the field of urology. The difficulty has increased with the introduction of the laparoscopic or robotic method.^[1]^ Early (within postoperative 90 days) complication rates after radical cystectomy range from 20% to 57%.^[2–5]^

Among the diversion methods, continent cutaneous diversion by Indiana or Kock pouch creation serves as an alternative option for patients in whom continence is desired in the setting of a nonfunctional urethra or a positive intraoperative urethral margin precluding the ability to perform orthotopic diversion.^[6]^ Myers et al had reported that perioperative complications after Indiana pouch creation or similar continent diversion may be complicated by wound infection (12%–33%), bowel leakage (1%–6%), and ureteral stricture (1%–21%).^[7]^ However, internal herniation caused by ureteral adhesion is extremely rare. We report the first case, to our knowledge, of small bowel obstruction and internal herniation occurring between bilateral ureters and urinary diversion after the procedure of robot-assisted radical cystectomy (RARC). Both patients have provided informed consent for publication of the cases.

## 2 Case presentation

### 2.1 Case 1

**Figure 1:**
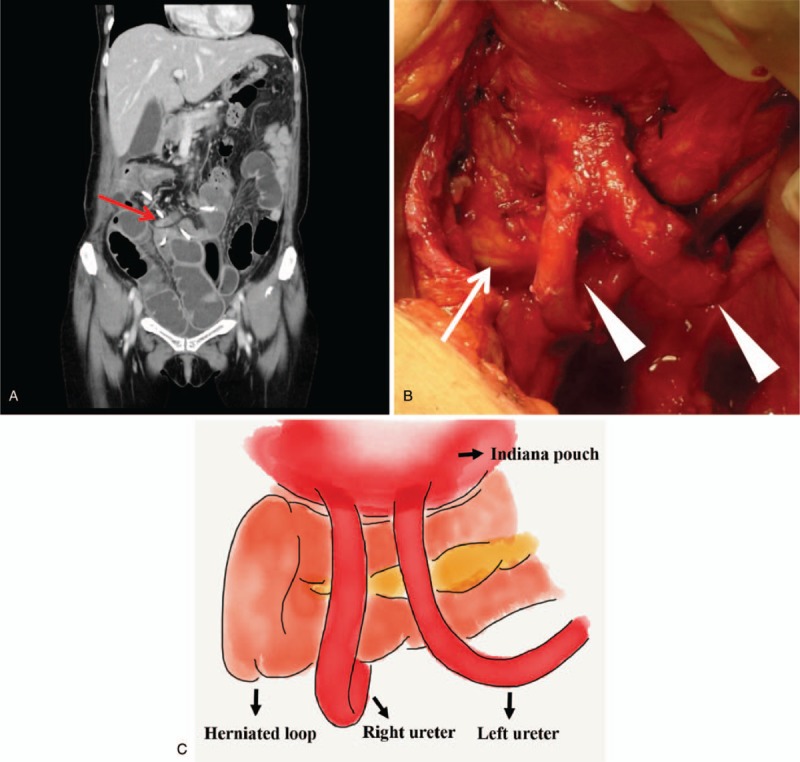
(A) Abdominal CT showed herniated loop between bilateral ureters and caused bowel obstruction (arrow: herniated bowel segment; arrowhead: ureter). (B) Bowel segment herniation and adhesion between bilateral ureters and pouch identified during laparotomy. (C) Sketch of the relative position of the bilateral ureter, urinary diversion, and the hernia loop.

A 64-year-old woman had undergone RARC with Indiana pouch creation for urinary bladder urothelial carcinoma (UBUC). The Indiana pouch construction was performed extracorporeally with an 8-cm incision from the extended robotic trocar wound. Her left ureter passed underneath the sigmoid mesentery as a standard procedure. She recovered well after the procedure. However, on the postoperative day 38, she presented to the emergency department with chief complaints of fever and left flank soreness. Her body temperature was 38.9 °C. Physical examination revealed left costovertebral angle knocking tenderness. Laboratory test revealed leukocytosis (12,500/μL) and elevated C-reactive protein (14.28 mg/dL). Urine analysis revealed pyuria (208/μL). Abdominal computed tomography (CT) showed bilateral hydronephrosis, periureteral fat stranding, and pouch bowel wall swelling. She underwent bilateral percutaneous nephrostomy catheter insertion for obstructive uropathy and received antibiotic treatment for acute pyelonephritis. Twenty days later, she developed aggravated abdominal distention, nausea, vomiting, and constipation. Abdominal radiography revealed a distended bowel loop consistent with bowel obstruction. Abdominal CT demonstrated an obstruction ileus with a distal intestinal transition zone and internal herniation between bilateral ureters and Indiana pouch (Fig. 1A).

During laparotomy, bilateral ureteroenterostomy sites were first identified, and then 1 segment of the ileal herniation adhered between bilateral ureters was detected (Fig. 1B and C). After careful dissection, manual reduction of the herniated bowel loop was performed. No ischemia or gangrene formation of the herniated bowel segment was noted. The postoperative course was unremarkable.

### 2.2 Case 2

**Figure 2:**
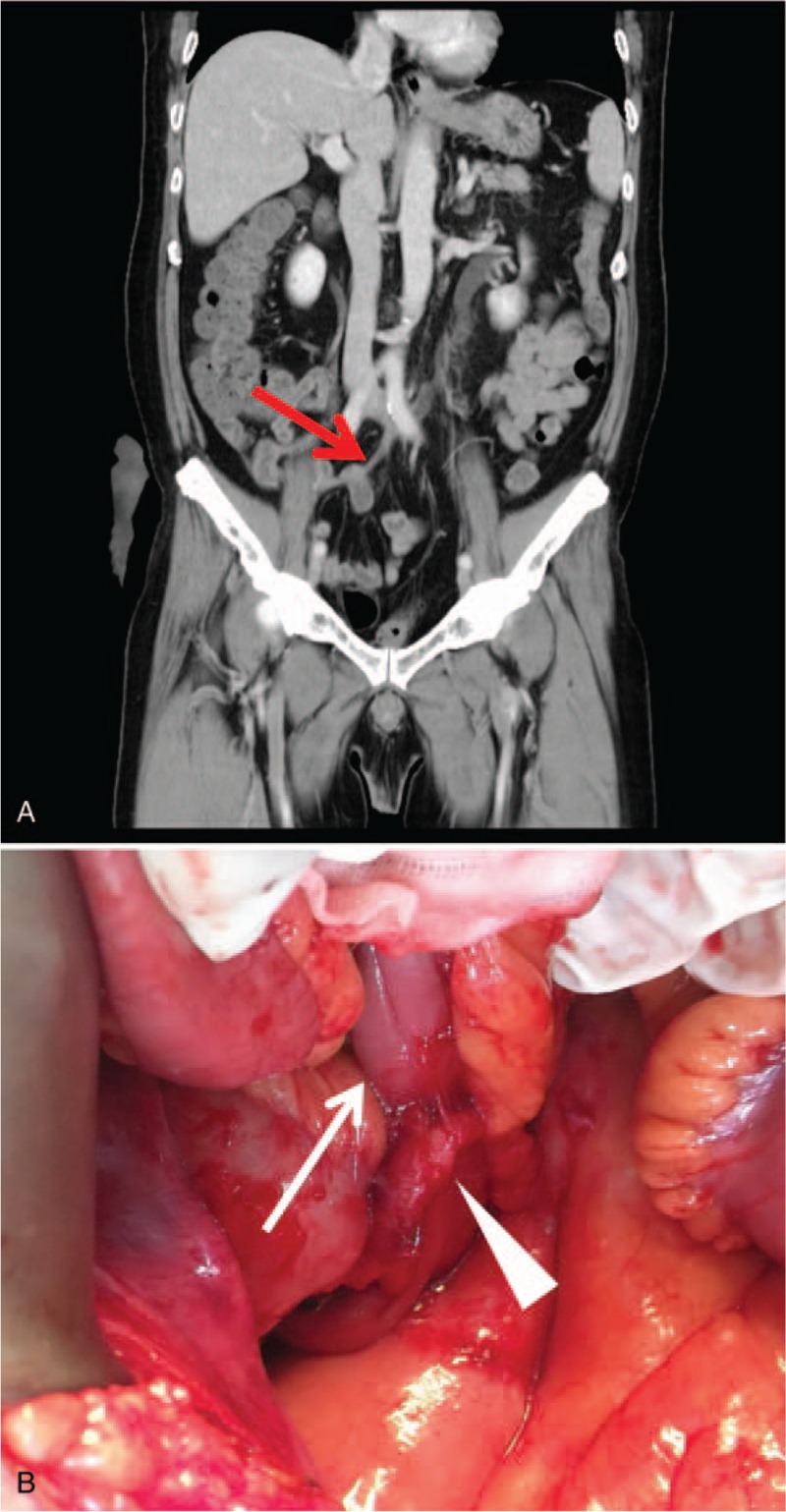
(A) Abdominal CT revealed 1 ureter stricture segment that caused hydronephrosis and hydroureter (arrow: ureter stricture). (B) During exploratory laparotomy, a segment of herniated bowel loop between bilateral ureters was found. The left ureter was complete surrounded by the small bowel and severe adhesion was noted. After complete ureterolysis, a 10Fr nasogastric tube could pass through the ureter smoothly. (Arrow: hernia loop, arrowhead: left ureter).

Another 61-year-old man underwent RARC with ileal conduit for UBUC. He experienced left pyelonephritis postoperatively. Abdominal CT 30 days after the surgery revealed left distal ureteral stricture with hydroureteronephrosis. The stricture was around 4 cm between the peritoneal window beneath the sigmoid colon and the ureteroenteric anastomosis site (Fig. 2A). Flexible ureterorenoscopy from the percutaneous nephrostomy showed no papillary tumor, but external compression of ureteral lumen was found. Urine cytology also showed a negative result for malignancy.

Due to recurrent pyelonephritis, he underwent open ureterolysis 6 months after RARC. During the procedure, we noted a small bowel loop herniating from the space between bilateral ureters and the conduit. The ureteral stricture was surrounded by the herniated loop, and adhesion between the ureter and intestine was observed (Fig. 2B). After sufficient ureterolysis and reduction of the herniation, a 10Fr. nasogastric tube could easily pass through the stricture. Paralytic ileus developed after ureterolysis and was treated supportively. His condition gradually improved, and he was discharged smoothly.

## 3 Discussion

Small bowel obstruction resulting from internal herniation between ureters and urinary diversion is extremely rare after radical cystectomy. Only 2 similar cases have been reported in the literature.^[8,9]^ To the best of our knowledge, this is the first case report of small bowel obstruction and internal herniation between ureters and urinary diversion after RARC.

**Figure 3:**
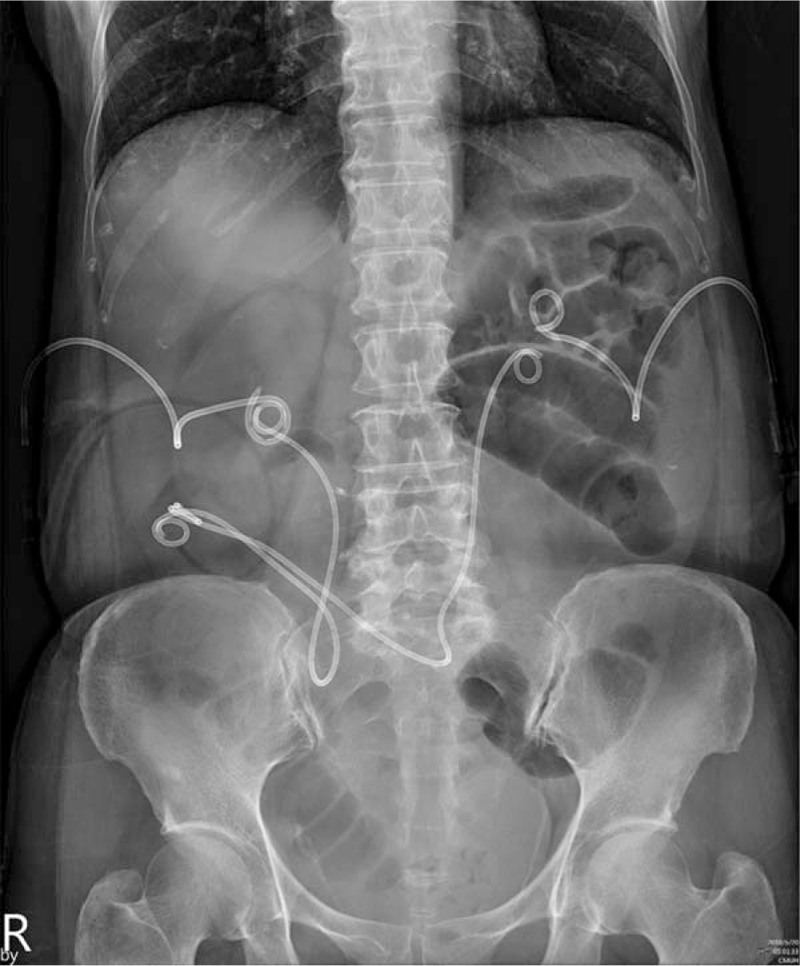
Abdominal X-ray of the patients showed a distended small bowel. Because of the high position of the stoma site, the bilateral ureters formed a U-shaped bend and lead to strangulation of the hernia loop.

Three factors may contribute to this rare complication. First, for cosmetic purposes we used the robotic trocar on the patient's right side as the position of the diversion stoma, rather than performing another incision.^[10]^ This trocar position is at the umbilicus level which is much higher than the usual position. The tented pouch/conduit and ureters, therefore, acted as a “hang rope” for the herniated segment. Abdominal X-ray of the case 1 patient showed a distended small bowel. Because of the high position of the stoma site, the bilateral ureters formed a U-shaped bend and lead to strangulation of the hernia loop (Fig. 3). Second, to facilitate extracorporeal diversion, we dissected and mobilized longer ureters than that in the open method. The redundant ureters and pouch/conduit created a vast space for a potential herniation. In the case 1 patients, we mobilized bilateral ureters to upper third level to facilitate the Indiana pouch construction process and to adjust the higher stoma position. In fact, the whole Indiana pouch position moved upward than previous experience due to the diversion outlet. During the laparotomy of case 2 patient, we found the ureter stricture segment was complete surrounded and fixed by a long small bowel loop herniated from the space created by redundant ureters and diversion. Third, urologists usually preform retroperitonealization during open radical cystectomy. However, the procedure is performed less often during laparoscopic or robot-assisted operation because of increased difficulty and limited operative field. Regaining normal anatomy of retroperitoneal space should decrease the incidence of internal hernia.

With the growing use of RARC for UBUC, the complication of small bowel internal herniation beside ureters may be more common in the future. Suitable stoma position, appropriate length of ureteral dissection, and retroperitonealization can help prevent such a catastrophic morbidity.

## Author contributions

**Conceptualization:** Chao-Hsiang Chang.

**Investigation:** Wei-Juan Li, Guang-Heng Chen.

**Supervision:** Chao-Hsiang Chang.

**Writing – original draft:** Li-Hsien Tsai.

**Writing – review & editing:** Po-Fan Hsieh.
